# Factors modulating maternofetal transfer of IgG antibodies following SARS-CoV-2 gestational infection

**DOI:** 10.1590/S1678-9946202567029

**Published:** 2025-04-14

**Authors:** Aline Almeida Bentes, Vivian Mara Gonçalves de Oliveira Azevedo, Stela Maris Aguiar Lemos, Gabriela Soutto Mayor Assumpção Pinheiro, Isadora de Araújo Martins, Nicholas Henrique Silva Cotta, Rafaela Martins dos Santos Oliveira, Gabriela Lousado Mesquita, Gabriela Cintra Januário, José Nélio Januário, Anuraj H. Shankar, Claudia Regina Lindgren Alves

**Affiliations:** 1 Universidade Federal de Minas Gerais, Faculdade de Medicina, Departamento de Pediatria, Belo Horizonte, Minas Gerais, Brazil; 2 Universidade Federal de Uberlândia, Escola de Educação Física e Fisioterapia, Uberlândia, Minas Gerais, Brazil; 3 Universidade Federal de Minas Gerais, Faculdade de Medicina, Departamento de Fonoaudiologia, Belo Horizonte, Minas Gerais, Brazil; 4 Universidade Federal de Minas Gerais, Faculdade de Medicina, Programa de Pós-Graduação em Ciências da Saúde - Saúde da Criança e do Adolescente, Belo Horizonte, Minas Gerais, Brazil; 5 Universidade Federal de Minas Gerais, Faculdade de Medicina, Belo Horizonte, Minas Gerais, Brazil; 6 Secretaria de Saúde do Estado de Minas Gerais, Belo Horizonte, Minas Gerais, Brazil; 7 Universidade Federal de Minas Gerais, Faculdade de Medicina, Departamento de Clínica Médica, Núcleo de Ações e Pesquisa em Apoio Diagnóstico, Belo Horizonte, Minas Gerais, Brazil; 8 University of Oxford, Nuffield Department of Medicine, Centre for Tropical Medicine and Global Health, Oxford, United Kingdom; 9 Oxford University, Clinical Research Unit, Jakarta, Indonesia

**Keywords:** SARS-CoV-2, Antibody transplacental transfer, Antibody persistence, Newborn, Modulators

## Abstract

Early infant immunity to SARS-CoV-2 depends on maternofetal transfer of antibodies. We aimed to analyze the factors modulating the maternofetal transfer of anti-SARS-CoV-2 IgG antibodies following gestational infection during the pandemic in Brazil (April–August 2021). We conducted a retrospective and prospective cohort study involving 509 mother-child dyads tested simultaneously for IgG anti-nucleocapsid antibodies during universal neonatal screening. There were 341 seronegative dyads and 168 seropositive ones. Seropositive neonates were retested two to three months later. We examined the association of neonatal serological status and IgG concentrations with gestational mRNA vaccination, timing of maternal infection, neonatal conditions, and gender. Gestational SARS-CoV-2 infection predicted neonatal IgG seropositivity (OR=3.97; 95%CI=2.69–5.88). Maternal infection in the first, second, or third trimester was associated with progressively greater seropositivity in neonates (34.4%, 51.6%, and 58.2%, respectively; p=0.03). Among seropositive neonates, IgG concentration was higher when mothers reported they had COVID-19 during pregnancy (p=0.04) and tended to be lower in girls (p=0.06). More than half of the seropositive neonates remained seropositive two to three months later (54.1%), which was associated with both maternal and neonatal IgG concentration at birth (p<0.001). Higher neonatal IgG concentrations at birth were associated with the persistence of anti-N IgG antibodies for two to three months in more than half of the seropositive newborns. This study provides an additional understanding of the dynamics of maternofetal antibody transfer.

## INTRODUCTION

Infant immunity to SARS-CoV-2 in the first months of life depends strongly on the maternofetal transfer of IgG antibodies during pregnancy^1–7^. Although previous studies have investigated the transplacental transfer of anti-SARS-CoV-2 antibodies, there is a lack of consistent data on factors that affect the dynamics of infection-induced antibody transfer and persistence^3–6,8–12^.

SARS-CoV-2 infection induces the production of IgG antibodies to spike (S) protein, followed by antibodies to the nucleocapsid (N) proteins one to two weeks later in most individuals^5,8,13^. Moreover, the type of antibodies induced by SARS-CoV-2 immunization varies depending on the vaccine used^9,14,15^. For instance, mRNA vaccines induce anti-S antibody production but do not induce an anti-N response. Thus, the presence of anti-N antibodies in people vaccinated with mRNA vaccines can discern previous or breakthrough SARS-CoV-2 infections, unlike anti-S, which is induced by both infection and vaccination^12,16–18^. Generally, the mean half-lives for anti-N are assumed to be 85 days, and for anti-S, 180 days^8,9,13,18^.

A recent study involving healthcare providers in a hospital during the first two years of the pandemic found that only 4.8% of people with a positive PCR result were seronegative for anti-N IgG. Meanwhile, 27.3% of the professionals who were seropositive for anti-N were negative by PCR, and 35.2% of the individuals who tested positive by either PCR or anti-N IgG were asymptomatic^18^. Similar findings were reported by Madewell *et al.* ^19^ in Puerto Rico, with 25% of the participants showing anti-N seroconversion without a positive RT-PCR result, probably due to asymptomatic or mild infections. The authors highlight anti-N antibodies as an optimal serological marker of Sars-CoV-2^18,19^. During pregnancy, maternal antibodies are produced following symptomatic or asymptomatic SARS-CoV-2 infection, and the antibody production positively correlates with maternal viral load^5,6,10,11,20,21^.

The intrauterine transfer of maternal IgG following COVID-19 has been reported to increase with time from infection to childbirth^2,3,5,6,11,22–25^. Moreover, the ratio of antibody concentration in the newborn compared to that of the mother, the transfer ratio, has been reported to be inversely correlated with the maternal viral load during pregnancy^10^, suggesting that more severe infections may adversely affect intrauterine transfer. Indeed, placental injury, immunity, and inflammatory processes in infected pregnant women may reduce maternofetal antibody transfer^7,12^. Although other authors have shown that mothers diagnosed with severe COVID-19 symptoms had a higher transfer ratio^26^, higher and longer lasting maternal antibody concentrations than those asymptomatic or mildly symptomatic^21^, recent studies did not find differences between symptomatic and asymptomatic mothers regarding the transplacental IgG transfer ratio^5,24,25^.

In addition, antibody concentration and transfer ratio may be sex-specific, as two studies found that the anti-SARS-CoV-2 transfer ratio measured in cord blood was lower in male neonates than in females^24,27^. Despite the potential protection provided by natural SARS-CoV-2 infection during pregnancy, maternally transferred IgG concentration may drop quickly during the first months after birth, thus providing limited protection for infants^25,26,28^ and possibly more for mothers who got infected in the early stages of pregnancy^29^.

Sero-surveillance using dried blood spots (DBS) collected from neonates at birth may be useful for estimating gestational exposure in pregnant women, especially given the high proportion of asymptomatic or unconfirmed infections^9,14,16,30^. Damjanovic *et al*.^9^ showed that anti-N seroprevalence in newborn DBS samples is strongly correlated with COVID-19 case incidence, while anti-S prevalence is correlated with vaccination coverage among women of reproductive age. The authors also demonstrated that perinatal conditions, such as low birth weight, multiple births, gestational age, and maternal characteristics, such as maternal age, modified the likelihood of a neonate being seropositive at birth^9^. Moat *et al*.^30^ also highlight surveys using DBS samples obtained from the newborn screening may contribute to monitoring pregnant women's immunity to SARS-CoV-2, both from vaccination or natural infection, and enable interventions to be developed to reduce gaps in protective immunity in pregnant women. Morshed *et al*.^14^ also claim that DBS is an attractive alternative for conducting serological surveys since most studies have shown high agreement between the results of samples collected by DBS and venous blood and because it is a very accessible technique.

Despite advances in knowledge, the dynamics of transplacental antibody transfer following COVID-19 infection during pregnancy are poorly understood, and many divergent findings persist^7^. Thus, this study aimed to analyze factors modulating the maternofetal transfer of anti-SARS-CoV-2 IgG antibodies following gestational infection and assess gestational vaccination, timing of maternal infection, gestational age at birth, birth weight, and gender as modulators of the maternofetal transfer, as well as of the persistence of the antibodies acquired by the fetus during gestation.

## MATERIALS AND METHODS

### Study design and setting

We conducted a retrospective and prospective longitudinal follow-up involving mother-child dyads from five municipalities in the southeastern region of Brazil. The municipalities were chosen based on the monthly number of live births and the rate of SARS-CoV-2 infection in the general population in December 2020. The dyads were recruited between April and August 2021^31^. During the recruitment period, the health professionals who were responsible for collecting DBS for neonatal screening invited all mothers who attended with their neonate in one of the 200 primary health centers involved in this study to participate in the serologic survey. Thus, if the neonates were accompanied by any other person instead of the mother, they were not eligible for the study.

### Participants and sample selection

**Figure 1:**
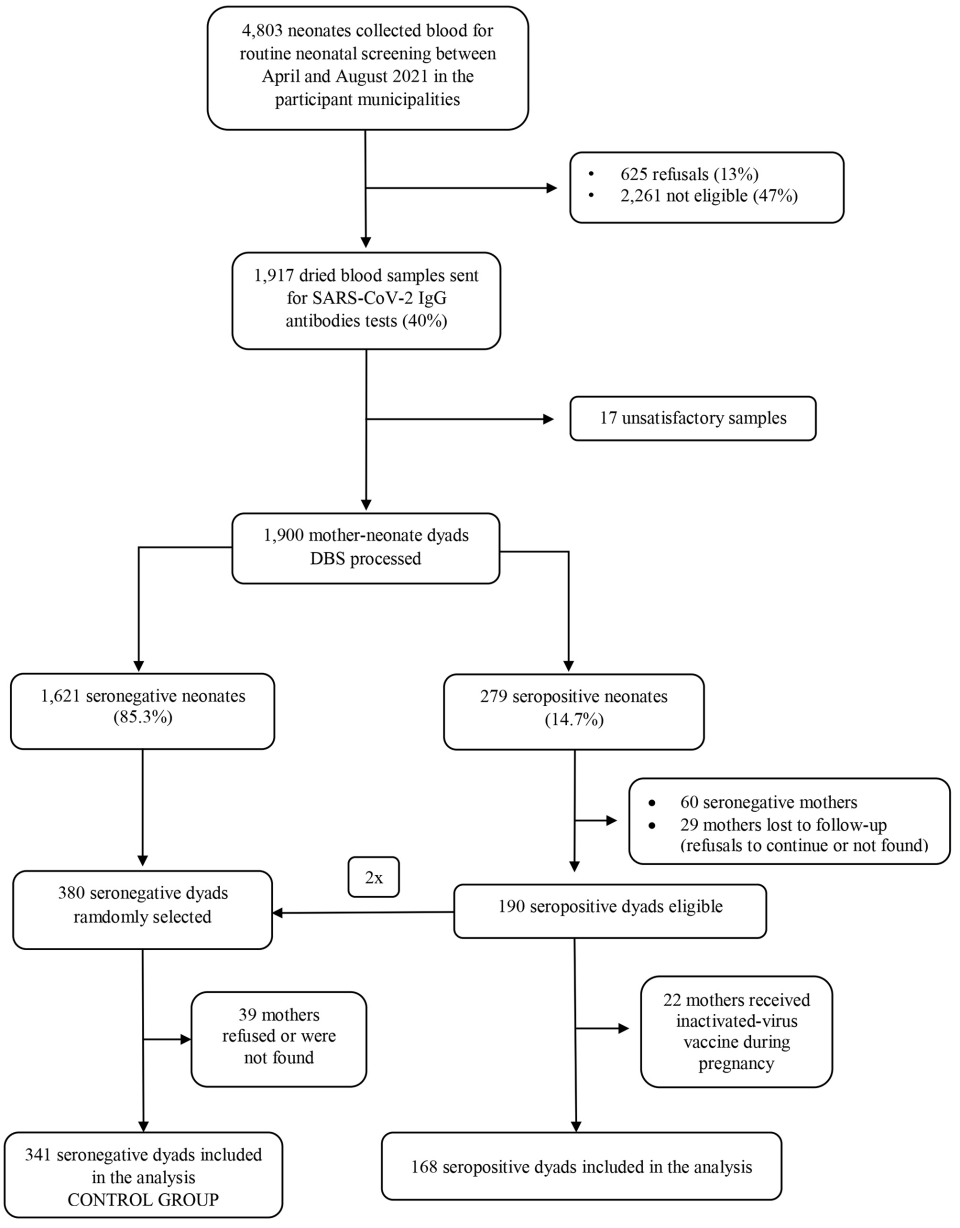
Study flow diagram.

We used non-probability sampling for serologic survey^31^. The inclusion criteria were any neonate who were up to 10 days of age and their mother who attended a public primary health care center for routine neonatal screening in the selected municipalities. Neonates diagnosed with COVID-19 before neonatal screening were excluded. During recruitment, finger-prick blood spots from 4,803 newborns were collected for neonatal screening in the participating municipalities according to procedures from the National Newborn Screening Program (NNSP) (Figure 1). Four samples from neonates who were older than 10 days at the time of DBS collection were excluded to reduce the likelihood of seroconversion due to postnatal infection.

### Study size

Overall, 1,900 dyads met the inclusion criteria and had DBS tested for IgG antibodies against SARS-CoV-2. Among the 1,900 dyads tested, 245 (12.9%) mothers and 279 (14.7%) neonates were seropositive^17^. Sixty seronegative mothers of seropositive neonates were removed from the sample, and another 29 seropositive dyads were lost to follow-up because their mothers could not be contacted in the first month after birth or refused to continue in the study. Figure 1 shows the study flow diagram.

Among the 190 seropositive dyads, 22 mothers were excluded from the analysis because they had received the inactivated virus vaccine during pregnancy (CoronaVac^®^), which elicits anti-nucleocapsid (anti-N) IgG antibodies, like natural infection, thereby confounding the ability to discern breakthrough infections from the vaccine response. In contrast, we retained mothers who received messenger RNA (mRNA) vaccines (Comirnaty^®^, AstraZeneca^®^), which elicit only anti-S IgG^17,32^, thereby enabling us to attribute the subsequent presence of anti-N antibodies to breakthrough infections. Due to the initial vaccination protocols of the Ministry of Health of Brazil, only pregnant women in the third gestational trimester could receive the first dose of mRNA vaccines.

The control group comprised 380 dyads, which were randomly selected from the initial 1,900 dyads and were seronegative for both mother and child, representing a control group that was twice the seropositive dyads (n=190). There were 39 seronegative dyads lost to follow-up whose mothers refused to continue in the study or could not be contacted in the first month after birth. Therefore, the final sample for analysis comprised 509 dyads (341 seronegative and 168 seropositive mother-child dyads).

### Data sources/measurement

Healthcare providers invited the mothers to join the study during routine neonatal screening, usually in the first week after birth. The neonates’ blood was collected onto filter paper by heel puncture following National Newborn Screening Program (NNSP) procedures. The mothers’ blood was collected on filter paper by finger-prick concomitantly with their children. The blood spots were dried and processed at the Center for Newborn Screening and Genetic Diagnostics of the Faculdade de Medicina of the UFMG (NUPAD).

The DBSs were processed by Enzyme-Linked Immunosorbent Assay (ELISA) using an EIA COVID-19 IgG-Dried Blood Spot^®^ test (Allserum Corp, China). The test on filter paper showed an almost perfect agreement (Kappa=0.915) with two tests on serum, using the same methodology (ELISA), with different protein epitopes (not published, internal laboratory report). The maternal vaccination status was heterogeneous among the mothers participating in the study, considering the type of vaccines, number of doses, and timing of maternal immunization, so we focused on anti-N transplacental transference as a marker of gestational infection. Furthermore, although DBS testing has already been validated to measure IgG antibodies against N and SARS-CoV-2 proteins^9,16^, anti-N ELISA kits were the only available ones for large-scale testing for anti-SARS-CoV-2 antibodies in DBS in Brazil at the time of the study.

Anti-N antibody concentration was quantified, which enabled maternofetal antibody transfer rates to be estimated. Neonates who tested seropositive in the first DBS were tested again with the same assay at two to three months of age to assess antibody persistence. All results were entered into an electronic system used by NUPAD for the NNSP.

Seropositive and seronegative mothers were interviewed by phone as soon as the serologic results were available in the NUPAD system, usually in the first month after birth. Answers were entered into a Google Forms questionnaire to record sociodemographic characteristics, immunization history, and gestational, perinatal, and laboratory data. None of the mothers reported being COVID-19 symptomatic at the time or right before the DBS collection, nor did their neonates appear to have any symptoms.

### Variables

The variables analyzed were: maternal age (< 20 years, ≥ 20 years); maternal years of schooling (< 9 years, 9-11 years, > 11 years and post-graduate); gender (female and male), gestational age (< 37 weeks, ≥ 37 weeks), birth weight (< 2,500g, ≥ 2,500 g), COVID-19 mRNA vaccine during pregnancy (yes, no), trimester of the SARS-CoV-2 infection (first, second, or third), maternal anti-N IgG concentration (log), neonatal anti-IgG concentration (log), and maternal and neonatal serological status (seropositive, seronegative).

The Socioeconomic Status (SES) was measured using the Criteria for Economic Classification of the Brazilian Association of Market Research^33^, which measures the purchasing power of families. The families’ socioeconomic status was categorized as high-medium (A and B levels), low (C level), and very low (D and E level).

The mothers were classified as infected if they had COVID-19 diagnosed by a physician or a positive RT-PCR for SARS-CoV-2 during pregnancy. For those mothers, the trimester of the gestational infection was based on the date they were diagnosed with/tested for COVID-19.

### Statistical methods

The serologic results were imported daily from the NUPAD electronic data system and merged with the Google Forms data from the phone interviews. Research assistants weekly verified the consistency of the dataset.

Assessment of the maternal and neonatal anti-N IgG concentrations yielded a large Kolmogorov-Smirnov statistic, indicating the values were not normally distributed (p<0.05). Thus, anti-N concentrations were log-transformed (log[Conc]), which reduced the Kolmogorov-Smirnov statistic, indicating the transformed values were likely to be suitable as dependent variables for linear regression.

The association between neonatal serologic status (seropositive or seronegative) and binary predictors was examined using the Odds Ratio (OR) with 95% confidence interval (95%CI) and chi-square for trend for non-binary predictors (significance level 5%). We used the same procedure to examine the predictors of antibody persistence in the second sample for the seropositive neonates at birth.

We performed a multiple linear regression using the neonatal IgG concentration (log) as the primary dependent variable. The initial regression model included the maternal age and years of schooling, families’ SES, child gender, gestational age, birth weight, history of mRNA vaccination, and SARS-CoV-2 maternal infection during pregnancy as the independent predictor variables. Stepwise backward regression was used with the final model retaining all variables with p-value < 0.05.

Violin plots were used to display differences in the independent predictors of neonatal IgG concentration (log). The log antibody concentration means were compared using a t-test (significance level 5%). Analyses were conducted in EpiInfo 7.2.5.0 and R Studio.

### Ethics

The professionals read a standardized folder informing the mothers of the procedures, risks, and benefits of participating in the study. Following this, the mothers provided verbally informed consent for themselves and their neonates. The study and the verbally informed consent procedure were approved by the Research Ethics Committee of the Universidade Federal de Minas Gerais (CAAE N° 42269021.9.0000.5149).

## RESULTS

### Sample's characteristics

Table 1 shows the sociodemographic and clinical characteristics of the sample. The mothers were predominantly young women under 30 years old, with a small proportion being adolescents (5.7%). Most had more than 11 years of education (67.2%) and belonged to families with low or very low socioeconomic status (84.6%). During pregnancy, 23.6% of the mothers received mRNA COVID-19 vaccines, and 37.7% had confirmed or suspected having COVID-19 (n=192), with over half of these infections occurring in the third trimester (51%). The neonates were mostly born at term (96.4%), with adequate birth weight (≥2,500 grams; 97.0%), and the majority were male (53.9%).

**Table 1:** Sample's characteristics and predictors of neonatal serological status (bivariate analysis).

Predictor		N=509	Seronegative	Seropositive	OR
		(%)	(n=341)	(n=168)	(95%CI)
			n (%)	n (%)	
**Maternal age** (n=503), mean (SD) - *years*			28.6 (6.4)	29.0 (6.5)	
	< 20 years	27 (5.7)	17 (5.0)	10 (6.0)	0.83
	≥ 20 years	476 (94.3)	320 (95.0)	156 (94.0)	(0.34-1.92)
**Maternal years of schooling** (n=507)					0.53*
	<9 years	55 (10.8)	38 (11.2)	17 (10.1)	1.00
	9-11 years	111 (21.9)	66 (19.5)	45 (26.8)	1.52
	>11 years	244 (48.1)	170 (50.1)	74 (44.0)	0.97
	Post-graduate	97 (19.1)	65 (19.2)	32 (19.1)	1.10
**Families’ SES** ^33^					0.92*
	High-medium	99 (14.4)	69 (20.2)	30 (17.9)	1.00
	Low	322 (63.3)	211 (61.9)	111 (66.1)	1.21
	Very low	88 (17.3)	61 (17.9)	27 (16.0)	1.02
**Gender**	Female	235 (46.2)	160 (46.9)	75 (44.6)	1.10
	Male	274 (53.9)	181 (53.1)	93 (55.4)	(0.74-1.62)
**Gestational age,** (n=498), mean (SD) - *weeks*			38.8 (1.3)	38.9 (1.2)	
	< 37 weeks	18 (3.6)	14 (4.2)	4 (2.4)	1.75
	≥ 37 weeks	480 (96.4)	319 (95.8)	161 (97.6)	(0.60-6.32)
**Birth weight** (n=503), mean (SD) - *grams*			3,224.5 (415.3)	3,269.5 (468.9)	
	< 2,500 g	15 (3.0)	9 (2.7)	6 (3.6)	0.75
	≥ 2,500 g	488 (97.0)	326 (97.3)	162 (96.4)	(0.26-2.29)
**COVID-19 mRNA vaccine during pregnancy**	Yes	120 (23.6)	96 (28.2)	24 (14.3)	0.43
	No	389 (76.4)	245 (71.9)	144 (85.7)	(0.26-0.69)
**SARS-CoV-2 infection during pregnancy**	Yes	192 (37.7)	92 (27.0)	100 (59.5)	3.97
	No	317 (62.3)	249 (73.0)	68 (40.5)	(2.69-5.88)
**Gestational trimester of the SARS-CoV-2 infection** (n=192)					0.03*
	First	32 (16.7)	21 (22.8)	11 (11.0)	1.00
	Second	62 (32.3)	30 (32.6)	32 (32.0)	2.04
	Third	98 (51.0)	41 (44.6)	57 (57.0)	2.65

* Chi-square for trend; OR = Odds Ratio; 95%CI = 95% confidence interval; SD = Standard Deviation; SES = Socioeconomic status.

### Predictors of neonatal serological status

Table 1 shows the bivariate analysis considering the full sample (n=509 dyads). There were no differences between seropositive and seronegative groups regarding maternal sociodemographic characteristics or neonatal conditions. Notably, the likelihood of newborns being seropositive for anti-N IgG was significantly lower if their mothers had received mRNA vaccination compared to those whose mothers were not vaccinated (OR=0.43; 95%CI=0.26-0.69). In contrast, the likelihood of seropositivity was almost four times greater for neonates of women who suspected having SARS-CoV-2 infection compared to those of non-infected mothers (OR=3.97; 95%CI=2.69-5.88). We observed that 40.5% of mothers of seropositive neonates did not report suspected COVID-19 during pregnancy, and among the confirmed infected mothers, only 52% of the neonates were seropositive.

When considering only women who suspected having COVID-19 during pregnancy, neonates of women infected in the second and third trimester had 2.0 and 2.7 greater likelihood of being seropositive than those of women infected in the first trimester, respectively (p=0.03). The neonatal seropositivity rate was 34.4%, 51.6%, and 58.2% if the pregnant woman had been infected in the first, second, or third trimester, respectively.

### Predictors of the neonatal IgG concentration (log)

Table 2 shows the initial and final linear regression models analyzing the predictors of neonatal anti-N IgG concentration (log). Only seropositive neonates (n=168) were included in this analysis. The neonatal anti-N IgG concentration was significantly lower when no SARS-CoV-2 infection was suspected during pregnancy (p=0.04) and tended to be lower if the neonate was a girl (p=0.06).

**Table 2:** Multiple linear regression models for predictors of neonatal IgG concentration (log).

Predictor		Initial Model				Final Model			
		*b*	Sth Error	F-test	p-value	*b*	Sth Error	F-test	p-value
Maternal age (adolescent/adult)		-0.004	0.08	0.003	0.96				
Maternal schooling (ref. <9 years)	9-11 years	0.04	0.07	0.37	0.54				
	>11 years	0.07	0.06	1.08	0.30				
Post-graduate		0.12	0.07	2.82	0.09				
Families’ SES^33^ (ref. High-medium)	Low	0.03	0.05	0.32	0.57				
	Very low	0.09	0.07	2.05	0.15				
Gender (Male/Female)		0.06	0.04	2.62	0.11	0.06	0.03	3.57	0.06
Gestational age (term/preterm)		-0.07	0.15	0.26	0.61				
Birth weight (low/normal)		0.14	0.11	1.72	0.19				
COVID-19 vaccine (Yes/No)		-0.05	0.05	0.81	0.37				
SARS-CoV-2 infection during gestation (Yes/No)		0.07	0.04	4.39	0.04	0.07	0.03	4.36	0.04
		r^2^ = 0.10				r^2^ = 0.05			

Outcome = Neonatal IgG concentration (log); Sample = seropositive neonates (n=168); SES = Socioeconomic status; *b* = coefficient.

**Figure 2:**
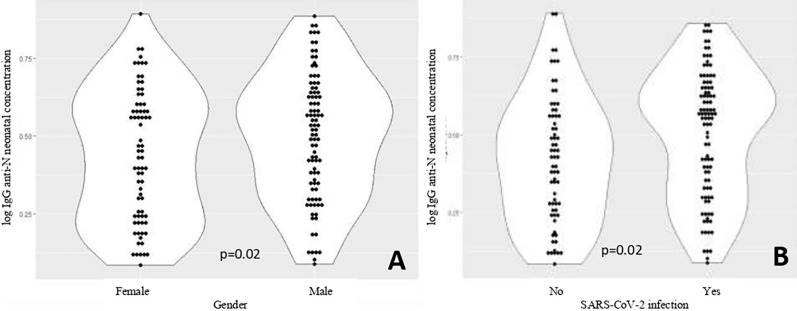
Violin plots showing neonatal IgG concentration (log) patterns by child gender and SARS-CoV-2 infection during pregnancy (n=168, seropositive neonates): (A) Neonatal IgG concentration (log) by child gender; (B) Neonatal IgG concentration (log) by maternal SARS-CoV-2 infection. SARS-CoV-2 = severe acute respiratory syndrome coronavirus 2; IgG = immunoglobulin.

Figure 2 shows the violin plots and comparative analysis of neonatal IgG concentration according to the gender of the child and suspected maternal infection. The neonatal IgG concentration (log) means tended to be lower in girls than boys (violin plot A; p=0.02) and in children whose mothers were not suspected of having SARS-CoV-2 infection during pregnancy (violin plot B; p=0.02).

### Antibody persistence

Among the 168 initially seropositive neonates, we obtained a second DBS from 159 infants (95%) between two and three months after birth (mean age=69.7 days; SD=11.2). Of these, 54.1% (n=86) had detectable antibody titers. The seropositivity in the second sample was associated with maternal IgG concentrations (log) (Means [SD]: seronegative=0.30[0.19] *vs.* seropositive=0.50[0.22]; p<0.001) and neonatal IgG concentrations (log) in the first sample (Means [SD]: seronegative=0.33[0.17] *vs*. seropositive=0.52[0.17]; p<0.001). No other variables were associated with antibody persistence (Table 3).

**Table 3:** Predictors of anti-N IgG antibody persistence from birth to 2–3 months of age.

Predictor	N=159	Seronegative in the 2^nd^ sample	Seropositive in the 2^nd^ sample	OR (95%CI)
		(n=73)	(n=84)	
		n (%)	n (%)	
**Maternal age** *				
< 20 years	9	5	4	1.47
≥ 20 years	148	68	80	(0.38-5.70)
**Maternal years of schooling**				0.16**
<9 years	17	7	10	1
9-11 years	42	26	16	2.32
>11 years	70	29	41	1.01
Post-graduate	30	11	19	0.83
**Families**’ **SES** ^33^				0.28**
High-medium	27	10	17	1
Low	107	50	57	1.49
Very low	25	13	12	1.84
**Gender**				
Female	69	35	34	1.41
Male	90	38	52	(0.75-2.65)
**Gestational age** *				
< 37 weeks	4	2	2	1.24
≥ 37 weeks	152	68	84	(0.17-8.99)
**Birth weight**				
< 2,500 g	6	3	3	1.19
≥ 2,500 g	153	70	83	(0.23-6.06)
**COVID-19 mRNA vaccine during pregnancy**				
Yes	22	14	8	2.31
No	137	59	78	(0.91-5.88)
**SARS-CoV-2 infection during pregnancy**				
Yes	94	40	54	0.72
No	65	33	32	(0.38-1.36)
**Trimester of the SARS-CoV-2 infection** (n=94)				0.25**
First	10	5	5	1
Second	30	15	15	1
Third	54	20	34	0.59
**Maternal IgG concentrations (log)**				
Means [SD]		0.30[0.19]	0.50[0.22]	<0.001***
**Neonatal IgG concentrations (log)**				
Means [SD]		0.33[0.17]	0.52[0.17]	<0.001***

Sample = seropositive neonates (initial sample =168; seven lost to follow-up: final sample = 159);

* missing data; OR = Odds Ratio; 95%CI = 95% confidence interval;

** p-value: Chi-square for trend;

*** T test; SES = Socioeconomic status.

## DISCUSSION

This study analyzed the factors modulating neonatal anti-N IgG serologic status and concentration in the first week after birth. Suspect SARS-CoV-2 infection during pregnancy predicted both. Furthermore, children who were born from mothers who had received mRNA vaccination against SARS-CoV-2 during pregnancy were less likely to have detectable anti-N antibodies a few days after birth than those whose mothers were not vaccinated. Female neonates tended to have lower anti-N IgG concentrations than males. Maternofetal antibody transfer rates in seropositive neonates progressively increased for confirmed or suspected COVID-19 from the first to the third trimester. More than half of neonates who were seropositive in the first 10 days after birth remained so for 60-90 days after birth. Antibody persistence was predicted by maternal and neonatal anti-N IgG concentration at the initial assessment.

Maternal age and schooling, family SES, gender, gestational age, and birth weight were not associated with neonatal anti-N IgG seropositivity, as reported in most previous studies^1,2,4,10,11,23,25^. However, Damjanovic *et al*.^9^ reported that low birth weight, multiple births, and maternal age above 30 years were associated with lower seroprevalence of neonatal antibodies against SARS-CoV-2. Additionally, Brebant *et al*.^24^ found that the transfer ratio was positively associated with later gestational age at delivery. In our sample, half of the neonates whose mothers had suspected SARS-CoV-2 infections during pregnancy were anti-N IgG seropositive, similar to previous studies reporting neonatal and umbilical cord seropositivity after infection during pregnancy (47-59%)^5,10,20,22,26^. However, other authors have shown higher transplacental antibody transfer rates in symptomatic pregnant women^5,6,11,12,23–25,34^. Vercoutere *et al*.^6^ analyzed a large multicenter Belgian cohort of unvaccinated mothers and found that four out of five mothers transferred antibodies to their neonates (81.3%) and the younger the gestational age at maternal infection, the higher the placental transfer ratio. Enengl *et al*.^5^ found that the percentage of IgG seropositive neonates born to infected mothers increased significantly, from 17.1% to 76.4%, over a six-week period following maternal infection, suggesting the timing between maternal infection and serological testing may influence the maternofetal transfer of antibodies. These findings can somehow explain the difference between the studies that only measure antibody concentrations once.

In our sample, most pregnant women were asymptomatic or had mild symptoms, and none reported COVID-19 symptoms at the moment of the blood collection. Still, in agreement with previous studies^5,6,34^, the suspect cases of gestational infection predicted the neonatal anti-N IgG concentration. Nevertheless, Song *et al.* ^26^ showed that severe and critically ill pregnant women had a transfer ratio significantly higher than asymptomatic or mild to moderately symptomatic mothers. In our study, mothers with confirmed infection had mild symptoms, which may be related to the presence of antibodies in only 52% of the babies. However, a large study with pregnant women who had similar clinical profiles found more than 80% of seropositivity among neonates born from seropositive mothers^6^. Moreover, Vercoutere *et al*.^6^ found that the chance of transferring antibodies was 3.8 higher among symptomatic pregnant women than among asymptomatic ones.

Atyeo *et al*.^1^ and Vigil-Vasquez *et al*.^25^ found that the antibody transfer in mothers with active disease at childbirth was lower than in mothers who recovered from the infection. Factors such as FcRn expression, IgG subclass, antigen structure, IgG glycosylation, and maternal hypergammaglobulinemia might be related to the specific transfer of SARS-CoV-2 antibodies^1,6,7,24^. Placental function injury^7,12^ and high viral load^1,10^ are reported as compromising maternal antibody transfer, although other authors showed that maternal transplacental antibody transfer did not differ significantly by maternal disease severity^5,11,22^. Whether the severity of COVID-19 during pregnancy affects transplacental antibody transfer is still controversial.

The data are equivocal for the association between SARS-CoV-2 infection-to-delivery interval and maternal antibody transfer.^4^ Based on clinical diagnosis and RT-PCR results for COVID-19, our findings agreed with Vercoutere *et al*.^6^ and Zhang *et al.* ^23^, indicating higher transfer rates when the mothers were infected in the second and third gestational trimesters than in the first. Similar findings were described by Song *et al*.^26^, who showed higher transplacental IgG transfer when mothers were infected 60-180 days before childbirth. In contrast, many studies showed that antibody transfer ratios and concentrations increase with the increasing infection-to-delivery interval^2,3,5,6,11,22,24,25^. Nevertheless, Vercoutere *et al*.^6^ found that neonates born to mothers who got infected less than four weeks before delivery had lower IgG concentrations and reduced placental transfer rates compared to those born to mothers who got infected more than four weeks before delivery. Yet, others did not find a significant change in the transplacental transfer ratio with increasing infection-to-delivery interval^10,29,34^. Notably, the mean infection-to-delivery intervals reported in most previous studies were usually short or limited to the third trimester. In this study, as described in the literature^18–20,25^, many anti-N seropositive mothers (40%) had no COVID-19-related symptoms, making it hard to establish the exact moment of the gestational infection.

Maternal vaccination during pregnancy also predicted neonatal serological status. According to Brazilian protocols, pregnant women started receiving mRNA vaccines after June 2021. Thus, most mothers included in this study were in the third trimester and received only one vaccine dose before delivery^13^. Despite this, the chance of the newborns from vaccinated mothers being anti-N IgG seropositive was lower than mothers who were not vaccinated. A multicenter study also found that anti-N IgG concentration was significantly lower in cord blood among women vaccinated during pregnancy than in women who were naturally infected before childbirth^2^. A recent systematic review and meta-analysis found mRNA vaccination during pregnancy significantly reduced COVID-19 cases^15^. Specifically, vaccination reduced symptomatic cases by 78%, severe cases by 72%, and virologically confirmed SARS-CoV-2 infections by 82%.^15^ Since mRNA vaccines do not induce anti-N production, our findings are probably indirect evidence of vaccine protection, as demonstrated by recent systematic reviews^15,17,32^.

Among seropositive neonates, the anti-N IgG concentration tended to be higher in boys than girls. Differently, Otero *et al*.^21^ did not find differences in antibody concentration or transfer ratio by fetal sex. Nevertheless, Brebant *et al.* ^24^ found that being a male newborn was negatively associated with the transplacental antibody transfer and highlighted that there were no significant differences for other pathogens concerning neonate sex. Bordt *et al.* ^27^ also found that female newborns showed a significantly higher concentration of all anti-SARS-CoV-2 IgG subclasses in cord blood when 38 women who got infected with SARS-CoV-2 during pregnancy were compared to 30 non-infected ones. Male children are known to be more vulnerable to perinatal infectious diseases. This may be explained by the maternal innate immune responses, leading to differential antibody transfer from the mother to male or female fetuses, thus providing different immunity levels to boys and girls^7,27^. Specifically, during SARS-CoV-2 infection, interferon responses have been implicated in worse outcomes for adult males and in compromised placental antiviral responses, possibly mediated by altered Fc receptor expression and antibody glycosylation^7,27^. Despite this evidence, our findings of decreased anti-N IgG in female neonates do not corroborate the results of Bordt *et al*.^27^. Clinical and methodological issues may explain this divergence.

Bordt *et al*.^27^ included a very small sample of infected mothers, and the mean infection-to-delivery interval was 36.5 days. In our sample, maternal infection occurred throughout pregnancy, notably in the second and third trimesters. Mothers included in this study were mostly asymptomatic. In contrast, in the study from Bordt *et al*.^27^, more than half of mothers were diagnosed with mild to moderate or severe COVID-19 at recruitment, which may have compromised their placental function^1,12,27^. Brebant *et al*.^24^ measured anti-N, anti-S, and neutralizing antibodies against SARS-CoV-2 and found a higher transfer ratio for anti-N (87.5%) compared to neutralizing antibodies (71,4%). The authors suggest exploring deeply how isotypes and a type of antibody glycosylation produced after infection may affect the immunity and the transplacental transfer ratio for many pathogens^24^. Although we analyzed only the anti-N IgG antibody class, previous studies have shown a good correlation between anti-N IgG intrauterine transfer and transfer of other antigen-specific IgG^22,27,29^; thus, the disparate results are probably not attributable to this aspect.

Finally, more than half of seropositive neonates still had detectable levels of anti-N antibodies two to three months after birth, a proportion higher than in previous studies^28,35^ but lower than described by Song *et al*.^26^, which is between five to 12 weeks (62%), and Vigil-Vasquez *et al*.^25^, which is two months after childbirth (66.7%). Our findings agree with many reports of a high correlation between maternal and neonatal IgG concentrations^5,6,25,26,29^, and that a high maternal antibody titer favors the transfer of antibodies to the fetus^11,25,34,35^. However, our study adds to the literature by showing the association of the maternal and neonatal IgG concentrations at birth with the persistence of transplacental antibodies for at least two months. Similarly, Song *et al*.^26^ demonstrated that the lower the IgG levels at birth, the faster children became seronegative. Despite this, we cannot discard the possibility of actively acquired antibodies between the first and second blood samples, as most of our infants were born during the peak of the second pandemic wave in Brazil. None of the infants had confirmed COVID-19 during the six-month follow-up, suggesting the transferred antibodies may have provided some protection to infants who were seropositive at birth.

This study has some limitations. First, not all mothers had an RT-PCR confirmation of the infection during pregnancy. Then, only patients who had severe COVID-19 symptoms were tested following Brazilian protocols. Thus, data on maternal infection was based on the mothers’ reports, laboratory results obtained from official health system records, and serology performed after delivery. Also, considering the half-life of antibodies induced by natural COVID-19 infection, mothers infected in the very early stages of pregnancy may have seroreverted as well as their newborn, representing possible false seronegative dyads.

Regarding strengths, the DBS from newborns was collected simultaneously with their mothers a few days after birth during the routine neonatal screening in a large serologic survey. This ensured that the antibodies detected in neonates were acquired transplacentally and not from a postnatal infection. We measured anti-N antibodies, enabling us to affirm that they came from a natural infection and not from maternal immunization. Furthermore, recruitment and blood collection linked to routine neonatal screening enabled us to analyze a large and diverse community sample independently of the gestational history of SARS-CoV-2 infection and, indirectly, to estimate the incidence of COVID-19 among pregnant women. Notably, our sample is larger than most studies cited in this article.

Our results provide an additional understanding of the dynamics of maternofetal antibody transfer. Moreover, considering the numerous inconsistencies in literature, more studies focusing on antibody dynamics and placental immune function are necessary to clarify the results.

## CONCLUSIONS

Our findings suggest the maternofetal transfer of IgG antibodies was modulated by three key factors: a history of COVID-19 during pregnancy, the trimester in which the SARS-CoV-2 infection occurred, and the gender of the neonate. Maternal COVID-19 history predicted both SARS-CoV-2 transplacental antibody transfer and neonatal anti-N concentration. Neonates exposed to SARS-CoV-2 during the second and third trimesters had a greater chance of being seropositive than those exposed in the first trimester. We found indirect evidence of maternal protection offered by mRNA vaccination during pregnancy, as it negatively predicted the rates of seropositive neonates. Child gender marginally predicted neonatal anti-N concentration, with male levels being higher than female ones. More than half of seropositive newborns still had detectable levels of anti-N antibodies in the second month of life, and this was positively associated with maternal and neonatal antibody concentrations in the first week after birth.
